# Sex-related differences in cardiovascular physiology and pathology: insights from cardiac imaging

**DOI:** 10.3389/fcvm.2026.1931498

**Published:** 2026-10-12

**Authors:** Vasiliki Tasouli-Drakou, Andrea Varias-Menor, Srijit Jana, Ryan Ng, Andrej Petronijevic, Jasmine K. Dugal, Christos Sarantopoulos, Buddhadeb Dawn

**Affiliations:** 1 Department of Internal Medicine, Kirk Kerkorian School of Medicine at UNLV, Las Vegas, NV, United States; 2 Department of Internal Medicine, Valley Health System, Las Vegas, NV, United States; 3 Department of Internal Medicine, University of Texas at Tyler, Tyler, TX, United States

**Keywords:** cardiac imaging, cardiovascular disease, cardiovascular physiology, sex differences, women's cardiovascular health

## Abstract

Although cardiovascular disease (CVD) is the leading cause of mortality in women, CVD in women has long been understudied and underdiagnosed. This is largely based on a misconception that hearts in women are similar to those in men with regard to physiological characteristics and physiological responses. Nonetheless, in an era characterized by rapid advances in diagnostic cardiac imaging modalities, there is a greater need to understand sex differences in cardiac anatomy and function, and how these might influence CVD patterns. Such differences include observations that women have greater left ventricular (LV) ejection fraction and more pronounced LV hypertrophy compared with men using echocardiography and magnetic resonance imaging (MRI) respectively. Imaging can help reveal microvascular dysfunction and heart failure, both of which have different characteristics in women compared with men. This review aims to provide a brief summary of the sex differences in cardiovascular pathophysiology among men and women observed across various imaging modalities, including echocardiography, MRI, coronary computed tomography angiography, positron emission tomography, and stress testing, while also referencing sex differences in electrophysiology with respect to cardiac anatomy.

## 1 Introduction

It is commonly known that cardiovascular diseases (CVD) in women are understudied and underdiagnosed, despite being their leading cause of mortality. Women are often undertreated, are more likely to be told that their symptoms are not cardiac, and in some cases, are less likely to receive angiography and vascularization procedures even after ST-segment elevation myocardial infarction (1). This is because of a common misconception that female hearts are similar to those in males. In addition, when using similar diagnostic criteria for female and male hearts, cardiac diseases in women are frequently overlooked by routine exams only to be diagnosed later at more severe stages (2). The historical underrepresentation of females in clinical studies and trials, including those that have shown that men and women equally benefit from an intervention such as intravascular imaging (IVI)-guided Percutaneous Coronary Intervention (PCI), may as a result lead to poor clinical outcomes, especially for structural devices or valves not made for or tested in adequate number of women (3, 4). To take it one step further, despite the fact that prevalence, age at diagnosis, and severity of symptoms of cardiomyopathies are sex-specific, diagnostic criteria for heart failure are not (5). Similarly, there is a paucity of sex-specific thresholds for quantitation of valve lesions and sex-specific imaging studies, despite the fact that women are more prone to having mitral and tricuspid valve diseases than men (6).

Cardiovascular differences between women and men exist at cellular, structural, and functional levels, and may reflect distinct physiological demands experienced across the life course. These differences range from female cardiomyocytes displaying greater resilience against stress-induced apoptosis compared with those from males, to women being more predisposed to certain pathological conditions such as prolonged QTc syndrome and heart failure with preserved ejection fraction (7, 8). It is conceivable that sex differences in cardiovascular physiology may have been shaped by the distinct reproductive demands experienced by females and males, as demonstrated by the increases in plasma volume and cardiac output that are observed in pregnant women (9). Nonetheless, biological roles are not sufficient to explain cardiovascular sexual differences, which are influenced by various additional factors, including but not limited to hormonal and environmental exposures (10).

While it is important to understand sex differences in symptom presentation, sex differences in diagnostic imaging should also not be ignored. For example, a study that examined men and women with traditional risk factors and with a low-intermediate Framingham risk using electron beam or multislice computed tomography (CT), found that although women were more likely to be older and had a higher coronary artery calcium (CAC) score compared with men, they exhibited a 1.44-fold higher 15-year adjusted mortality hazard (11). Likewise, advances in comprehensive diagnostics including coronary computed tomography angiography (CCTA) and magnetic resonance imaging (MRI) have revealed important differences. These include a higher prevalence of imaging-detected coronary atherosclerosis in men, as well as observations that female hearts beat at faster heart rates, produce larger contractile strains, and exhibit grater ejection fraction despite generating a smaller cardiac output (Figure 1) (2, 12). Thus, by taking into account sex-specific diagnostic differences, we can not only better understand how sex-specific cardiac anatomy and physiology influence the genesis and progression of cardiovascular diseases in men and women but also aim to produce a greater shift toward implementing guidelines that incorporate sex-specific thresholds. For these reasons, this review article seeks to summarize sex-specific differences observed across imaging and non-imaging diagnostic modalities that highlight why the female heart isn't just a smaller version of the male one.

**Figure 1:**
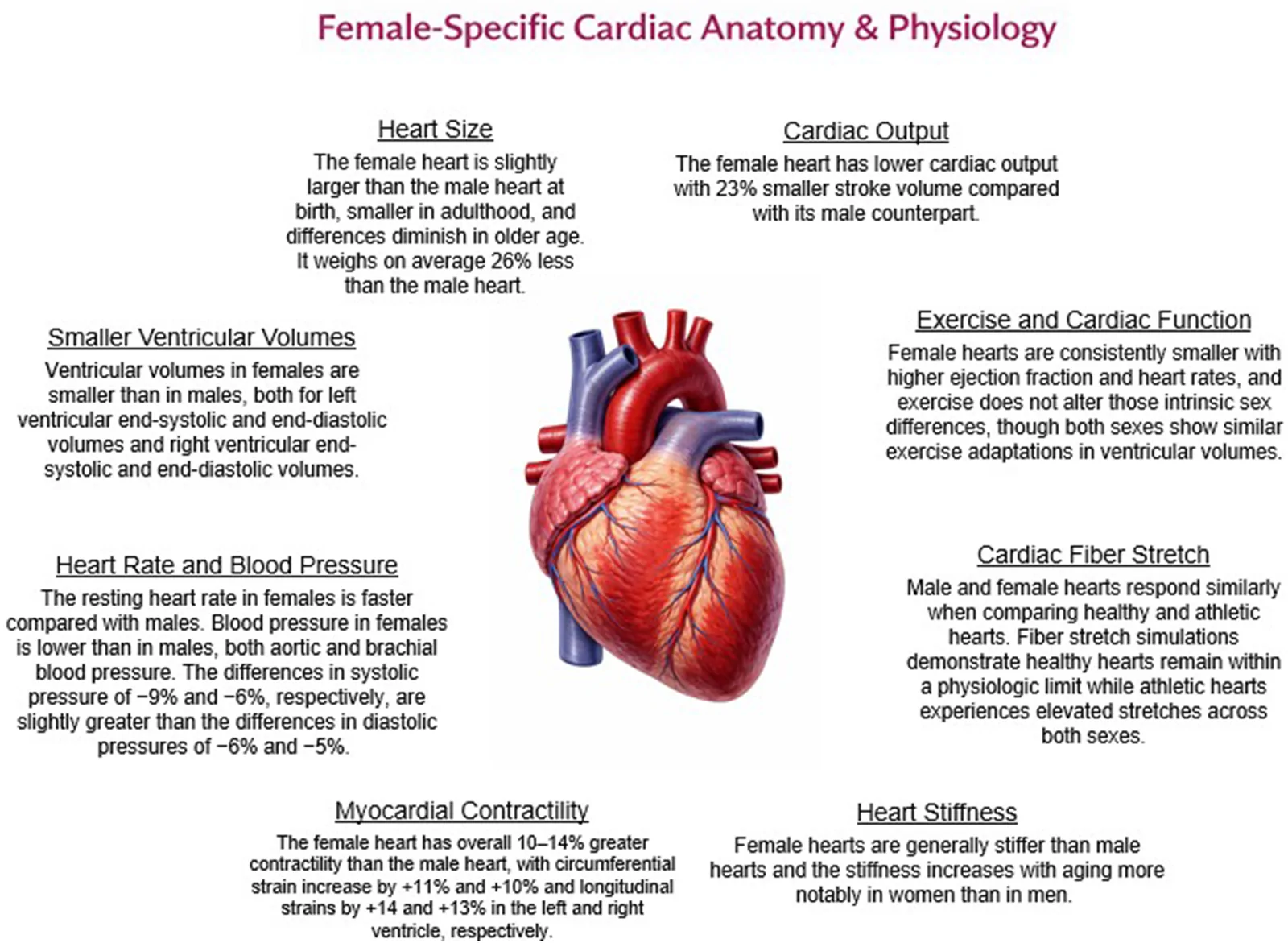
Sex-differences in cardiovascular anatomy, physiology, and pathobiology. Schematic representation of the heart highlighting sex-specific differences in cardiac structure, function, and physiology (2, 13, 14).

## 2 Sex-specific cardiac anatomical and structural differences

To examine differences in cardiovascular disease outcomes between males and females, it is important to first understand anatomical differences between the male and female hearts. At birth, a newborn female heart is about 5% larger in mass compared with a newborn male heart, but later during adulthood the pattern switches as the average adult male heart is 26% larger compared with the average adult female heart (2). In order to investigate the differences in cardiac mass, it is important to acknowledge the role of cardiomyocytes. Men and women are born with similar numbers of cardiomyocytes, but when measured later during adulthood, women are found to have a greater number of cardiomyocytes in their ventricles compared with men (Figure 2) (15). The increased percentage of cardiomyocytes within the ventricles of the female hearts is believed to account for the previously observed 10%–14% increased contractility in female hearts compared with male hearts (2). Some studies have attributed this difference in cardiomyocytes to the activation of pro-survival Akt signaling pathway, which in turn inhibits the initiator caspase-9 and the effector caspase-3 that are involved in cardiomyocyte apoptosis (16).

**Figure 2:**
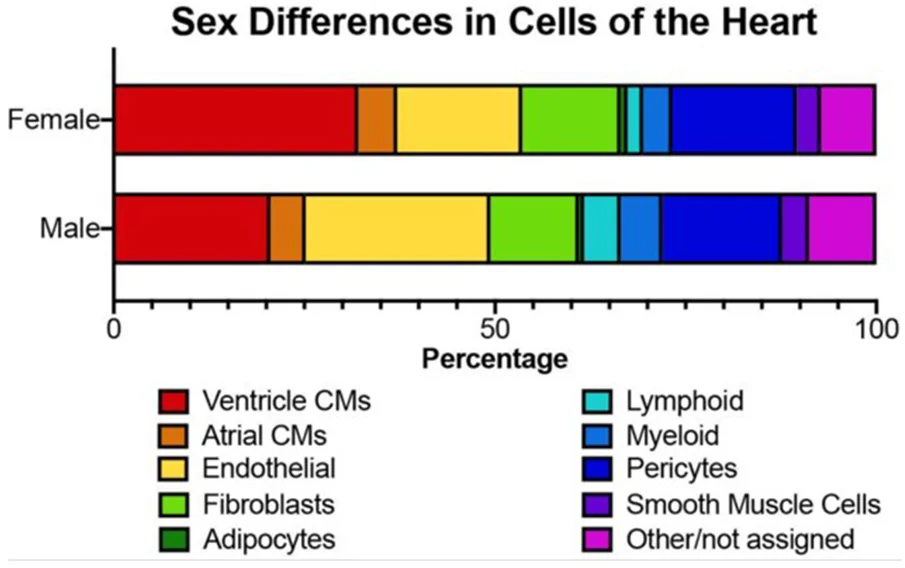
Sex differences in human cardiac cellular composition. Reproduced with permission from the Journal of Molecular and Cellular Cardiology. CM, cardiomyocyte (Copyright Clearance Center license number 6255030089289) (17).

Testosterone has been found to play a role in increasing myocardial contractility (18). This is due to testosterone binding to androgen-receptors which bind to the L-type calcium channels; this increased amount of calcium within cardiomyocytes allows for increased contractility (19). At lower levels of testosterone, cardiac contractile function has been shown to be reduced in addition to increased levels of oxidative stress within cardiac cells (20). On the other hand, at higher levels of testosterone (typically seen with anabolic steroid usage), testosterone has been seen to induce apoptosis of cardiomyocytes (7). Furthermore, sex also plays a significant role in apoptosis, as the apoptotic index is three times higher in men compared with women (21).

The difference in the presence of increased levels of cardiomyocytes in the ventricles of adult female hearts may also be attributed to differences in cellular protein expression, completely independent of the input of hormones. Shi et al. suggested that these differences in cardiomyocyte gene expression begin in early development, and drive differential expression of genes and proteins in male vs. female hearts (22). This suggests an effect on future cardiomyocyte proliferation. Their study further alleged that the specific cardiac protein alpha-1B-glycoprotein (A1BG), which is commonly found in both embryonic and adult female heart tissue, when disabled, leads to decreased contractile function and increases in cellular stress only in the female heart. This finding is also supported by a subsequent study by Emerson et al. who employed an A1BG-knockout method and found that A1BG is essential for the maintenance of ventricular structural integrity only in female hearts, leaving male hearts unaffected, further suggesting a possible relationship between specific sex-differentiated proteins and myocardial function (23).

Sex differences can also play a large role in cardiac remodeling and hypertrophy. The primary female sex hormone, estrogen, has been widely recognized as a cardioprotective hormone that prevents left ventricular (LV) hypertrophy in pre-menopausal women (24). Estrogen plays an important role in stimulating nitric oxide production by acting on a cellular receptor called endothelial estrogen receptor (ERa), further protecting against heart remodeling (25). Moreover, estrogen has also been found to prevent oxidative stress-induced endothelial cell apoptosis (26). However, female endothelial cells have been reported to show higher apoptosis and intracellular reactive oxygen species compared with male cells, potentially suggesting a more vulnerable endothelial phenotype in females (27). Endothelial cells form the lining of blood vessels that supply the heart and have been linked to induce apoptosis of cardiomyocytes as a result of TLR4-mediated NF-kB signaling (28). Estrogen levels, measured by the primary circulating form 17B-estradiol (E2), significantly fall during menopause, and the decrease in estrogen is associated with increased heart disease incidence and increased cardiac remodeling (13).

The adult male heart has greater mass in all chambers, larger chamber volumes, and cardiac output, despite the female heart having an increased ejection fraction and heart rate (2). While the male heart is larger in dimensions, it is believed that sex differences between male and female hearts can be attributed to differences in lean body mass and are also believed to apply linearly in every dimension of the heart. However echocardiographic studies have found that cardiac geometry does not scale linearly (29). In an echocardiographic study of 734 healthy men and women, Kou et al. found that adult males had a 30% greater LV mass but only an 11% difference in LV septum thickness and 9% higher posterior wall thickness compared to adult female hearts. Further, in an autopsy study analyzing 1,062 morphologically normal hearts using similar ages, body mass index (BMI), and body surface area (BSA), females had a significantly smaller heart mass than males (285 ± 55 g vs. 374 ± 64 g, in females and males, respectively, *p* < 0.001) (30). In addition, female hearts also had greater sub-epicardial fat compared with males. Lastly, Moscolini et al. used 3D statistical shape modeling (SSM) to characterize the geometry of the heart of 456 healthy subjects from a UK imaging biobank and reported that sex accounts for 25% of variability in cardiac anatomy even after adjusting for body size (31). This study revealed that female hearts had a reduced anterior-posterior width in the right ventricle and suggested that female hearts also have less elongated ventricles, indicating that the differences in mass between male and female hearts do not scale linearly with cardiac anatomic geometry.

## 3 Sex-specific differences in cardiac conduction and electrophysiology

Sex-specific differences in electrocardiography are receiving increasing attention in the field of cardiovascular medicine today. Despite its widespread use and the fact that electrocardiography is one of the most important and commonly used diagnostic tests in medicine, normal values for electrocardiograms (EKGs) are typically derived from men, and the normal physiological ranges for women have often not been fully appreciated. Studies show that female heart has distinct electrophysiologic properties as well as morphologic features that may influence conduction and repolarization, which in turn could play an important role in the development and maintenance of arrhythmias (32).

Sex related differences in EKG occur typically after puberty and seem to be influenced by hormones in the autonomic nervous system. It has been demonstrated that women have higher resting heart rates (HR) (about 5%–10%) in comparison with men, possibly as a result of different sympathetic activity levels, increasing vagal modulation, and/or changes in firing characteristics of the sinus node (32). Men also tend to have a longer PR interval, a wider QRS complex, and higher QRS voltages than women, while women have a shorter atrioventricular and intraventricular conduction times and longer QTc values (33, 34). Some of the sex-related differences in ventricular depolarization are due to differences in cardiac anatomy. Men tend to have larger ventricular dimensions and a higher amount of myocardial mass and thus larger QRS duration and amplitude (35). Aras et al. also found that normalization for body size eliminated some of the differences between male and female values with respect to the measured conduction intervals, thus providing further evidence for some sex-specific differences in ventricular electrophysiology (36).

Other major sex-related differences in EKGs relate to ventricular repolarization, ventricular action potential duration and QT intervals (37). These differences appear after puberty and may be the result of differences in hormonal effects on ion channels in atrial, nodal, and ventricular cells. Estrogen reduces repolarization reserve by blocking potassium currents, whereas testosterone shortens ventricular repolarization by decreasing calcium channel current and increasing potassium channel current; these mechanisms may be responsible for longer QTc values in women (37, 38). Women also are more prone to drug-induced QT prolongation, which may be a contributing factor in cases of torsade the pointes and other proarrhythmic effects. These are important considerations when choosing antiarrhythmic medications for women (38). It is important to note that despite the drop in estrogen, no significant QTc interval changes have been observed between menopausal and pre-menopausal women (39, 40). However, and interestingly, the differences in QTc intervals among the two sexes seem to decrease with increasing age, primarily in individuals greater than age 60 (39). This phenomenon is attributed to the declining testosterone levels in men, which in turn, leads to the decreased inhibition of L-type calcium currents and longer early repolarization.

Prolonged QTc is not the only proarrhythmic abnormality women are predisposed to. In general, the frequency of supraventricular tachycardias (SVTs) is twice as high in women compared with men. In addition, the stretching of atrial and ventricular myocytes by the increased plasma volume, the shortened refractoriness, and the high resting heart rate, all contribute to the higher prevalence of SVTs in pregnant women, primarily in the form of atrioventricular nodal reentrant tachycardia (AVNRT) and atrioventricular reciprocating tachycardia (AVRT) (9). The incidence of atrial fibrillation is lower in women compared with men, however, the absolute number of women with atrial fibrillation is greater than men because women tend to live longer, and the prevalence of atrial fibrillation increases with age (41). The degree of shortening of atrial effective refractory period (ERP) during atrial pacing was noted to be smaller in premenopausal women compared with men and postmenopausal women. Since shorter atrial ERP may promote atrial fibrillation, this observation may explain the increase in atrial fibrillation in postmenopausal women (42). With regard to additional sex-related differences in arrhythmias, men are more likely to have manifestations with accessory pathway-mediated arrhythmias, Brugada syndrome with consequent ventricular tachycardia and ventricular fibrillation (43). The presence of Brugada syndrome in men is attributed to the greater testosterone-mediated transient outward potassium current (Ito) which in turn leads to a greater action potential notch in the RV epicardium (44). Moreover, the burden of ischemic heart disease combined with the greater sarcoplasmic reticulum calcium leak in male cardiomyocytes may contribute to males' increased susceptibility to ventricular arrhythmias (45). Overall, the risk of sudden cardiac death is smaller in women compared with men (43).

Men also generally have higher ST-segment elevation and T-wave amplitude, whereas women tend to have smaller QRS amplitudes and ST-T voltages (32, 34). The above differences in ST-segment and T-wave morphologies may be related to the decreased sensitivity and specificity of exercise EKG in women, and these findings may also partly explain why the diagnosis of ischemic heart disease is often delayed in women, despite the fact that women may develop ischemic heart disease at older ages than men (34). Furthermore, sex-related EKG characteristics have also been described in the presence of atrial fibrillation. Laureanti et al. noted that the presence of atrial fibrillation does not eliminate the observed sex differences in the *P*-wave, as women have higher *P*-wave variability compared with men (46). This may indicate sex-specific differences in atrial remodeling in patients with atrial fibrillation, which may be related to sex-related differences in the burden of symptoms of atrial fibrillation and to the differences in atrial fibrillation-related adverse events. These observations along with other differences between male and female EKGs call into question the current single standard of reference for normal values for various EKG parameters. Increased awareness regarding sex-related differences in EKGs would suggest the formulation of sex-specific EKG criteria as part of individualized and more precise evaluation of data.

## 4 Sex-specific differences identified by echocardiography

Echocardiography, a radiation-free, non-invasive diagnostic test, is commonly used to detect structural abnormalities or valve disease and quantify cardiac size and function (47). Types of echocardiography differ by approach and include transthoracic echocardiography, stress echocardiography, transesophageal echocardiography, 3D echocardiography, and fetal echocardiography. Echocardiography has been used extensively because of its unique ability to provide real-time images of the heart and wide availability (48).

Echocardiography has also been used to differentiate cardiac characteristics between sexes. Specifically, when it comes to the left atrium, women generally have smaller volumes than men; however, in the context of co-morbid conditions including obesity and hypertension, age-related atrial enlargement is more prominent in women (49). Recent studies have shown that overweight women are more likely to present with left atrial dilatation compared with men with comparable BMIs, a finding that persists later in life (50, 51). These findings suggest that increased weight and obesity may serve as significant cardiovascular risk factors for women, predisposing to the development of subclinical cardiac disease.

With regard to ventricles, women have smaller LV and RV volumes than men when quantified by echocardiography (52). Importantly, these differences diminish when adjusted for lean body mass (53). Comparatively, men have larger absolute left ventricular end-diastolic volumes (LVEDV) than women, even after indexing to body surface area (52). These findings suggest that the observed sex-specific differences persist even when normalized for body size. A larger LVEDV in men, this may lead to their ability to achieve higher stroke volumes at different states of rest and exercise.

With regard to cardiac mass, women generally have reduced LV mass than men, after indexing to body surface area (52). LV mass when normalized to body size provides a standard measure known as the LV mass index (LVMI). In a retrospective study by Gebhard et al., 5,307 subjects were evaluated over a 21-year period using transthoracic two-dimensional echocardiography to review LVMMI compared in different age groups. LVMI increased in females from 66.4 ± 1.3 g/m^2^ between the ages of 7–20 years to 76.3 ± 0.9 g/m^2^ between the ages of 60–80 years (*p* < 0.0001) and in males from 81.9 ± 1.7 g/m^2^ between the ages of 7–20 years to 94.6 ± 1.3 g/m^2^ between the ages of 60–80 years (*p* < 0.0001). Overall, LVMI increased with advancing age, predominantly in females (54). With greater LV mass, women have more concentric remodeling, compared with men who have larger chamber dimensions and are more prone to develop eccentric LV hypertrophy (55).

With regard to LV systolic function, women typically have higher LV ejection fraction (LVEF) compared with men, even with certain pathologies, such as Heart failure with preserved ejection fraction (HFpEF), when age and body size are adjusted (Figure 3) (52, 56). In the same aforementioned study by Gebhard et al., LVEF and LV fractional shortening (LVFS) were also seen to increase with advancing age, particularly in females just as LVMI (54). A significant increase in LVEF was observed with age in both genders (*p* < 0.0001), with females showing an LVEF of 62 ± 0.5% between ages 7 to 20 years old and an LVEF of 65 ± 0.3% between the ages of 60 to 80 years, while males exhibited an LVEF of 62 ± 0.5% and 64 62 ± 0.3% for the respective age groups. LVFS represents the percentage decrease in the LV internal diameter from end-diastole to end-systole, thereby providing an additional measurement of cardiac contractility. In this study, LVFS increased in females from 37.7 ± 0.5% for those between the ages of 7 to 20 years old to 42.4 ± 0.4% for those between the ages of 60 to 80 years old (*p* < 0.001), while in males LVFS increased from 37.3 ± 0.5% to 39.4 ± 0.5% for the respective age groups (*p* < 0.001) (55).

**Figure 3:**
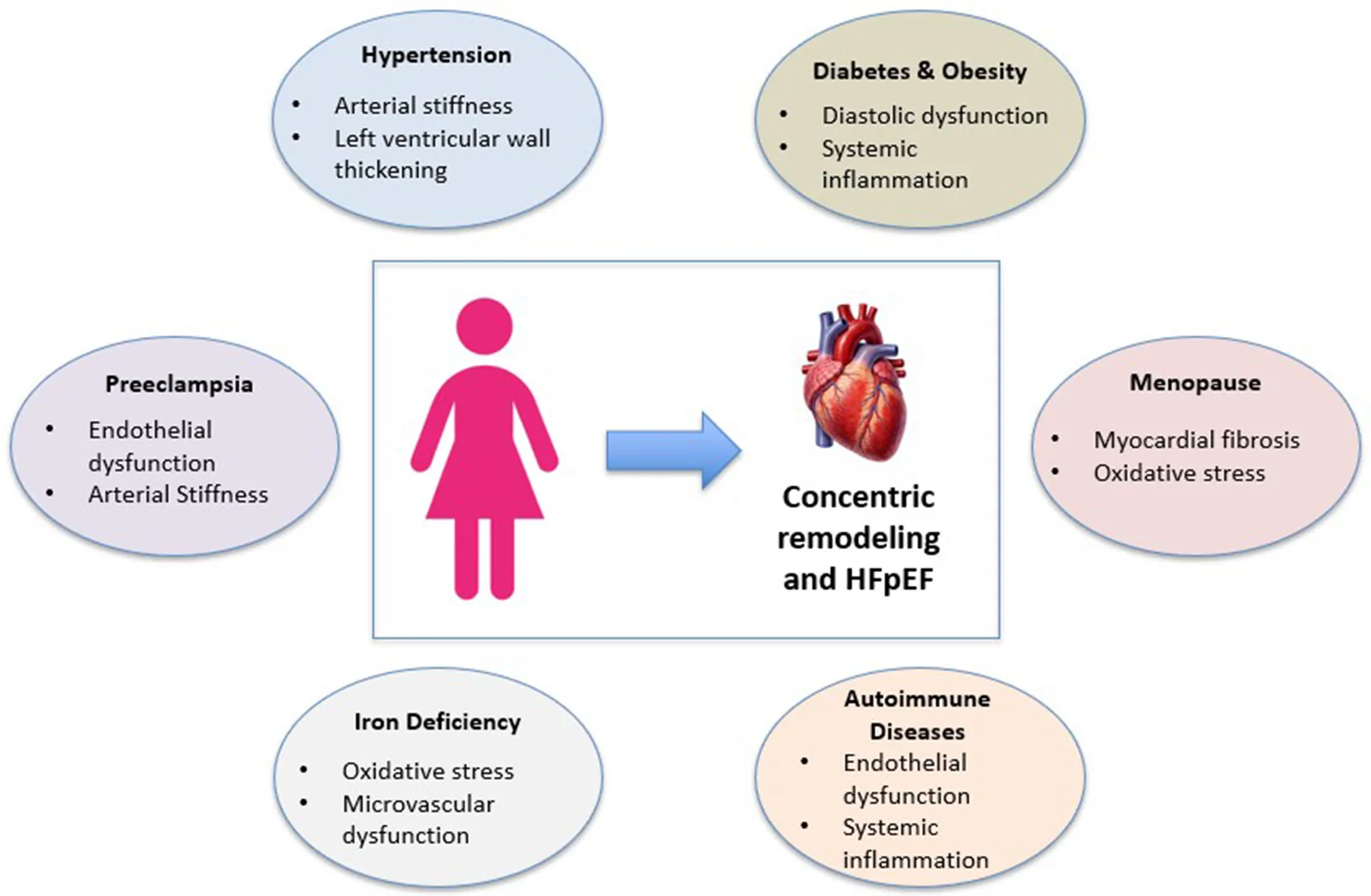
Graphic highlighting some of the underlying mechanisms that collectively predispose female hearts to concentric remodeling and the development of HFpEF (58).

LV diastolic function relates to the relaxation and filling phases of the heart. Studies have shown that women exhibit more pronounced diastolic dysfunction, in the form of impaired relaxation, resulting in elevated LV end-diastolic pressure (LVEDP) and higher E/e′ ratios both at rest or in exercise states (57). The LV end-diastolic volume index (LVEDVI) also decreases with age particularly in females, a finding that was confirmed by the study of by Gebhard et al. (from 49.8 ± 0.9 mL/m^2^ between the ages of 7 and 20 to 42.8 ± 0.6 mL/m^2^ between ages of 60 and 80, *p* < 0.0001) (54). With regard to HFpEF, data from 161 individuals with exertional intolerance and confirmed HFpEF revealed that females compared with males had higher pulmonary capillary wedge pressure (PCWP) indexed to peak exercise workload (58). In the same study, women were noted to have higher EF and smaller ventricular dimensions, suggesting that women exhibited smaller diastolic reserve and worse peripheral oxygen use compared with men. Indeed, alterations in diastolic function with advancing age are overall more pronounced in women (55, 56) (Figure 3).

These sex-specific differences identified in echocardiographic studies present an overall picture that women often have smaller cardiac chambers, different remodeling patterns, and different risks of pathology progression all necessitating sex-specific thresholds for severity assessment and timing for intervention (59). The observations with regard to LVEF, LVFS, LVMI, and LVEDVI indicate that with advancing age the LV undergoes life-long remodeling with patterns that are different in women compared with men. Nonetheless, there is still a lack of sex-specific cut-offs in LV diastolic function parameters that may lead to an underestimation of diastolic dysfunction in women, and this needs to be addressed (60). This is critical because sex differences in echocardiographic parameters have important prognostic implications, underscoring also the need for sex-specific thresholds for interventions, particularly when evaluations are based on echocardiographic findings.

## 5 Sex-specific differences identified by coronary computed tomography angiography (CCTA)

While women tend to have lower rates of coronary artery disease (CAD) compared with men, they tend to have worse outcomes and greater mortality necessitating accurate and precise risk stratification adjusting for differences between sexes (61). CCTA is a Class I indication for non-invasive screening for symptomatic CAD according to the American College of Cardiology/American Heart Association guidelines (62). Given that CAD is often underdiagnosed in women, CCTA serves as a diagnostic tool to bridge the difference in diagnosis between sexes. Women's risk of CAD is often underestimated by contemporary risk stratification scores such as the Framingham Risk Score and the Pooled Cohort Equations (63). While such scores take into account factors such as sex, cholesterol levels, race, history of smoking and hypertension, they fail to include other female-specific risk factors such as preeclampsia, gestational hypertension, gestational diabetes, and preterm delivery (64, 65). This is important as all of these missing factors are associated with manifestations of cardiovascular diseases later in life (66). In addition, the traditional risk factors may also have different effects in women and men. Diabetes, smoking, and hypertension have been shown to confer greater relative cardiovascular risks in women in several studies, contributing thus to the differences in risk assessment between the sexes (67). Therefore, a more complete and accurate assessment of CAD risk in women would need to incorporate and weigh both conventional and female-specific risk factors. Importantly, indeterminate exercise EKG results are more common in women due to nonspecific ST-T-wave changes which has led to an overuse of coronary angiography for invasive evaluation (68).

The CRESCENT Trial enrolled 350 patients, both males and females, from the Netherlands with stable chest pain, who were referred for the evaluation of possible obstructive CAD (68). Exclusion criteria included known CAD confirmed by stress test and/or previous coronary angiogram performed within the previous year. All patients underwent coronary artery calcium (CAC) scans; those with CAC scores between 1 and 400 or with a pretest probability of >70% underwent CCTA, while the rest, including those where CT was contraindicated, underwent stress EKG testing or invasive coronary angiography (ICA). The CCTA group was compared to the positive Stress EKG group with ≥1 mm ST Deviation. The primary endpoint was determined by absence of anginal symptoms at baseline and 1 year follow-up based on the Seattle Angina Questionnaire. Women had more atypical symptoms (57% vs. 46%, *p* = 0.032) and lower rates of obstructive CAD than men. Exercise EKG was associated with lower diagnostic accuracy and high rates of intermediate results in women. Compared with stress EKG, CCTA was associated with a more accurate and efficient diagnosis and staging of CAD in women requiring less downstream testing, including referrals for coronary angiograms This finding, however, could be attributed to patients being screened by CAC Scores for further evaluation of CAD.

Similarly, the PROMISE Trial, a randomized controlled trial, compared sex differences in results and prognostic information following CCTA and functional testing (i.e., exercise stress test, nuclear stress test, and stress echocardiography) (69). A total of 8,966 patients with symptoms suggestive of stable CAD, such as exertional chest pain, were enrolled, of which 4,500 patients (2,332 females) were randomized into the CCTA group and 4,466 (2,388 females) to the functional testing group. Women overall had lower rates of positive functional test and CCTA detecting significant (>70%) CAD. Women who had positive test results were less likely to be referred for coronary angiography for further management compared with men, and if they underwent angiography, they were less likely to have obstructive CAD. Finally, the trial found that over the span of the follow-up period (25 months), women who underwent non-invasive testing had better outcomes than men, alluding to the higher rates of completely normal coronary anatomy (69).

CCTA can be combined with Fractional Flow Reserve (FFR) assessment, otherwise known as FFR_CT_, to further evaluate the significance of coronary atherosclerotic burden. FFR_CT_ has been shown to better analyze and categorize coronary stenosis potentially more effectively than CCTA alone or functional testing. In a sub-analysis of the ADVANCE Registry, 4,738 patients (1,603 females) with symptomatic CAD, who had previous CCTA were further analyzed with FFR_CT_ (70). The inclusion criteria included CAD >30% degree stenosis on CCTA and age >18. Exclusion criteria included no CAD on CCTA, poor CCTA image quality, less than 1 year life expectancy, or non-compliance with follow-up. The results showed that women had less anatomically obstructive CAD on CCTA (≥50% stenosis) (65.4% vs. 74.7%; *p* < 0.0001) and severe stenosis (≥70% stenosis 27.1% vs. 34.9%; *p* < 0.001) than men who were more likely to have multivessel CAD. Women were also less likely to have positive FFR_CT_ ≤ 0.80 (*p* < 0.0001) for the same degree of CAD stenosis compared with men. Finally, the study observed that for women with a positive FFR_CT_ ≤0.80, they had equal referral rates for invasive coronary angiogram, but had lower rates of obstructive CAD and revascularization. As understanding of CAD in women evolves, more precise risk stratification frameworks are needed, particularly as CCTA emerges as an increasingly important tool in diagnostic evaluation and clinical decision-making. FFR_CT_ may thus serve as an additional adjunct to CCTA for CAD classification and management in women (71).

Furthermore, CCTA can be utilized to determine the severity of other pathologies besides CAD in women, proving to be increasingly versatile. Valvulopathies such as aortic stenosis (AS) can also be risk stratified using CCTA. Women often have less calcification of the aortic valve for the same degree of pathologic AS compared with men, which remains true even when adjusted for BSA. Calcium scores of 1,300 Agatston units (AU) and 2,000 AU are often used as the threshold to diagnose severe AS in women and men respectively (72). In addition, CCTA has been used for preoperative coronary evaluation in patients undergoing surgical or transcatheter aortic valve interventions (TAVI) given its good sensitivity and specificity in detecting significant CAD, thereby reducing the need for ICA (73). Moreover, when combined with FFR, FFR_CT_ had greater specificity in ruling out obstructive CAD in TAVI patients. Similarly, CCTA can be used for pre-operative (i.e., calcification, valvular thickening, subvalvular structure, and annular stenosis) and post-operative (i.e., annular remodeling and integrity of repair techniques) assessments of mitral valves in patients undergoing mitral valve repair (74). Nonetheless, while CCTA has better spatial resolution than echocardiography, it comes with a few limitations and drawbacks, including the use of radiation, especially at increasing doses to obtain a systolic phase; the use of medications to reduce heart rate and control arrhythmias; and finally, the lack of hemodynamic information that could easily be obtained using echocardiography.

In perimenopausal women, CAD risk can be further stratified by age given estrogen's cardioprotective ability. In a retrospective cohort study, Feuchtner et al. examined the diagnostic efficacy of CCTA in 1,882 patients (48.5% females) (75). When stratified by age and gender, women under the age of 47 had the highest rates of negative CCTA findings for coronary atherosclerosis (82.8%), with 4.6% of the group having findings suggestive of CAD. However, in women aged more than 50 years, the negative CCTA rates were markedly lower but still higher than men. In the same group, assessing for CAD with CCTA vs. CAC showed no statistical difference (negative CAC vs. CTA: 44.3% vs. 39.1%, *p* = 0.060). These observations were consistent with findings reported by Shaw et al., who examined sex differences in calcified plaque and mortality. Studies of CAC measures (number of lesions and vessels, lesion size, volume, and plaque density) showed that women had fewer calcified lesions compared with men and that CAC density was not predictive of cardiovascular mortality in women (76).

In summary, while the above findings suggest that women have a lower overall prevalence of CAD compared with men, they are still at a greater risk of adverse outcomes and overall mortality when it comes to ischemic heart disease (61). Given these discrepancies and the lack of reliability in contemporary risk scores, women with low to intermediate cardiovascular risks should be considered to undergo further screening, primarily with CCTA as well as CAC. When taking into consideration the findings in the CRESCENT trial, CAC can be utilized as an initial screening of CAD, particularly in premenopausal women (68). In both the PROMISE and the CRESCENT trials, assessing severity of coronary artery stenosis with CCTA prevented unnecessary downstream testing that was more likely to occur with routine functional testing such as exercise EKG or stress echocardiography, especially in combination with CAC scoring (68, 69). While CCTA is promising in terms of evaluating CAD and impacting the course of management in women, its growing versatility allows it to be potentially favorable in the assessment of valvulopathies as well, such as AS (77). In addition, promising adjunctive modalities such as FFR_CT_ aims to further assess the functional significance of the atherosclerotic burden in women when CAC and CCTA are indeterminate (71).

## 6 Sex-specific differences identified by cardiac MRI

Sex differences have also been identified in cardiac magnetic resonance (CMR) findings. In patients with hypertrophic cardiomyopathy (HCM) undergoing CMR, females were more likely to have smaller left ventricular (LV) end-systolic volume index, higher LVEF, and greater LV outflow tract (LVOT) acceleration flow, systolic anterior motion of the mitral valve and mitral regurgitation (MR), compared to male patients (78). These observations suggest that women with HCM are more prone to have obstructive physiology given their smaller left ventricular end-diastolic and end-systolic volumes. It is important to also note that female participants in this study were older and more symptomatic, alluding to the fact that women with HCM are diagnosed and treated later in life. Similar findings were also reported in the study by Shi et al. who noted that women tend to present with greater LV hypertrophy due to their susceptibility to increased LV load pressures (79). They also found that female subjects with concomitant diabetes and HFrEF exhibited more LV concentric remodeling, less impaired global systolic peak strain and a decreased likelihood of late gadolinium enhancement (LGE) presence, contributing to thus better survival. It is important to note given that LGE in cardiac MRIs suggest irreversible myocardial fibrosis or scarring (80).

CMR has also been used to investigate the relationship between body composition and cardiac structure and function in men and women. A cross-sectional study that analyzed over twenty-five thousand participants found that women exhibited a positive association between visceral adipose tissue volume (VAT) and LV mass and global mean myocardial wall thickness (MMWT) that was not observed in men, while appendicular lean mass (ALM) was associated with increased LV mass (LVM) and LV volumes in both women and men (81). They also demonstrated that when accounting for total body fat and lean mass, the association between VAT and LVM was attenuated in women. Similarly, when looking at cardiac muscle performance in young adults, studies analyzing cardiac 4D flow MRI findings found that although women had smaller LV volumes and peak systolic blood flow kinetic energy compared with men, they exhibited increased systolic and diastolic circumferential strain and blood flow vorticity, a finding that renders women's left ventricles more functionally efficient compared with men's due to more efficient flow and less energy dissipation (Figure 4) (82).

**Figure 4:**
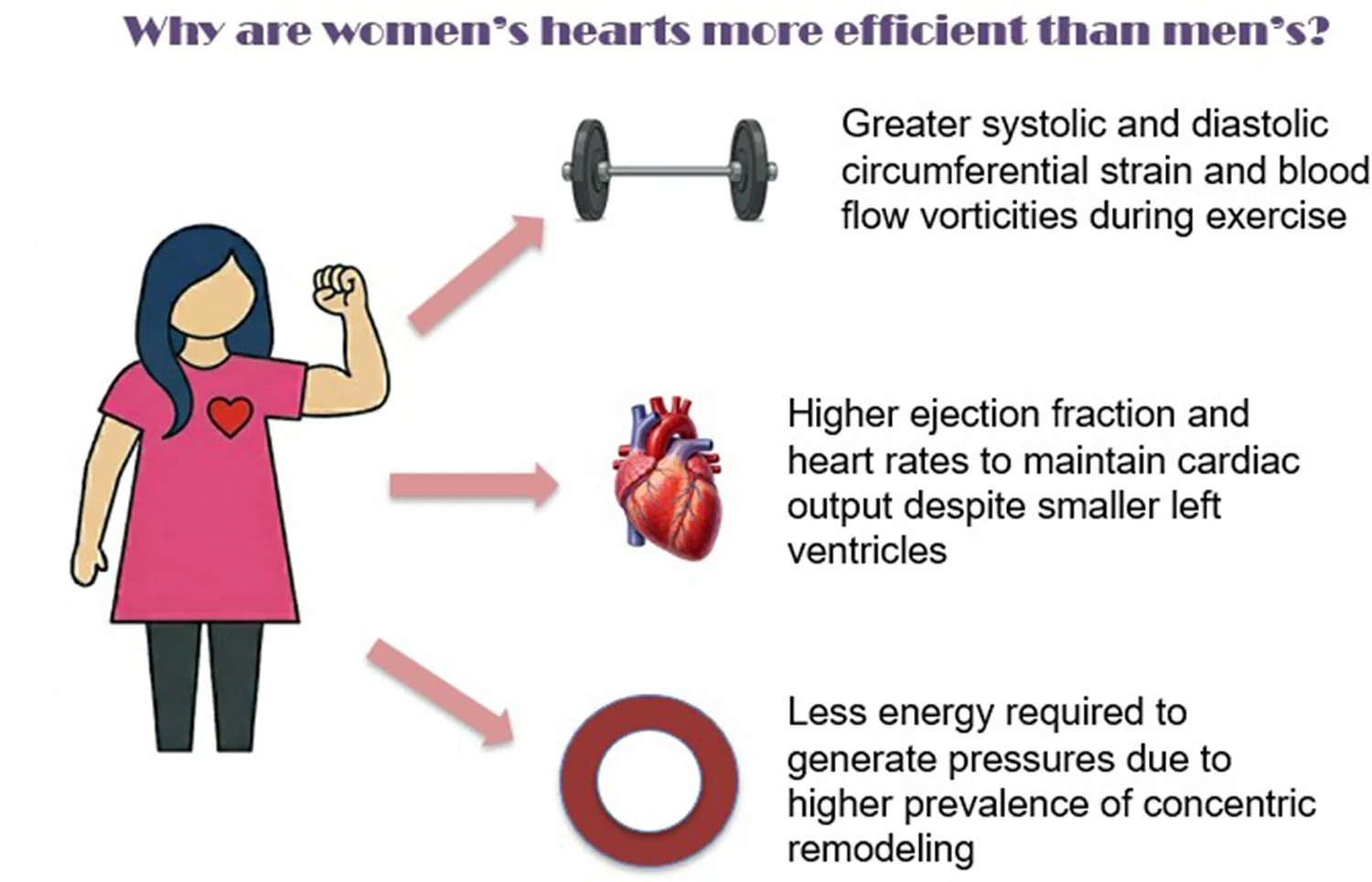
Figure showing that structural, functional and physiological differences identified using cardiac MRI indicate that women's hearts are more “efficient” than men's. These include favorable ventricular filling and pumping despite lower volumes, and less energy dissipation and more efficient fiber shortening during exercise.

It is important to note that CMR has also been used to better understand valvular disease in women in addition to echocardiography. As previously mentioned, women are more prone to suffering from mitral valve (MV) diseases such as MV prolapse (MVP) or rheumatic MV diseases compared with men. What makes CMR a superior tool to echocardiography is its ability to accurately quantify both MR severity and myocardial cavity and differentiate primary from secondary MR (83). Women have been found to have smaller regurgitant volumes than men with similar regurgitant fractions using CMR, suggesting that regurgitant volume thresholds for severe primary mitral insufficiency may be lower in women (84). This makes sense as women generally have smaller LV volumes and stroke volumes. Nonetheless, a recent study by House et al. found that the sex differences in regurgitant volumes secondary to MVP can be resolved by normalizing the regurgitant volume to the LV end-diastolic volume (85). This observation supports the need for sex-specific interpretation of imaging because using absolute regurgitant volume thresholds might underestimate MR severity in women by making clinicians perceive women's MVP severity as less severe, when in reality sex differences in regurgitant volumes are due to differences in heart size and not disease severity as such.

In a similar fashion, CMR has been used to understand sex differences in aortic regurgitation (AR) and aortic stenosis (AS). A CMR-based study by Kammerlander et al. found that in subjects with at least moderate AR on echocardiography, women in contrast to men frequently had a normal LV size (end-diastolic volume indexed to body surface area, LVEDV/BSA) despite severe AR, suggesting that the severity of chronic AR may be underestimated in female patients in the absence of LV dilation (86). Interestingly, another study by Malahfji et al. found that when adjusting for body surface area in patients with moderate or greater AR, indexed LVEDV and mass were not significantly different between sexes (87). With regard to AS, women are more predisposed to the development of low-flow low-gradient AS given its association with many predisposing factors that women have, including smaller stroke volume, LV concentric remodeling, smaller LV cavity, an increased relative wall thickness, a higher afterload, and diastolic dysfunction (88). Studies employing CMR have found that while women have greater diffuse and focal myocardial fibrosis independent of the degree of AS severity, they tend to progress to severe AS with lower aortic valve calcification levels in comparison with men (88, 89).

Finally, something that characterizes CMR is the variability in the various techniques used to characterize myocardial tissue. While T1 quantifies interstitial fibrosis by examining the extracellular fraction percentage of the myocardium, T2 and T2* mapping sequences are used to detect myocardial edema and iron overload, respectively (90). Using a 1.5 T CMR, Cadour et al. studied 175 healthy volunteers, and found that women exhibited greater T1 values compared with men. These values decreased with age, a difference that was more pronounced in females across all ages. In contrast, decreases in T2 and T2* values were not statistically different among sexes as age increased. Similar findings were reported in a study by Roy et al., who examined 75 healthy subjects aged 20 to 90 years old using 3 T CMR (91). The authors found that mean myocardial T1 and extracellular volumes unlike T2 values, were greater in age-matched women than in men, while T2* were not influenced by either age or sex. However, unlike the study by Cadour et al., there was a statistically significant increase in T1 values as age increased, especially among men, while no statistically significant change was observed in T1 values in women as age increased. These findings could help support the age-dependent increase in interstitial myocardial fibrosis observed in males. While there is little evidence in the literature supporting any clinical advantages of 3 T MRI studies over 1.5 T ones, a 2018 study by Min et al. found that 3 T CMR has superior diagnostic performance in patients with multi-vessel CAD without old myocardial infarctions (92, 93).

## 7 Sex-specific differences identified by positron emission tomography

Positron emission tomography (PET) has also been used to quantify and differentiate characteristics of cardiac anatomy between sexes. A study by Gill et al. found that with regard to pericardial adipose tissue (PAT), there was no significant sex difference in PAT between normal weight and overweight men and women. However, that PAT was significantly associated with higher glucose and triglyceride levels and lower HDL cholesterol levels in women, but not in men (94). The authors concluded that PAT is associated with an increased cardiometabolic risk (diastolic blood pressure, serum lipids, fasting glucose, abdominal subcutaneous and visceral adipose tissue) in women compared with men.

Moreover, PET myocardial perfusion imaging (MPI) has commonly been used to assess myocardial blood flow (MBF) and myocardial flow reserve (MFR). Importantly, among patients who underwent PET MPI, coronary small vessel disease (CMD) was more prevalent in females (95). The authors stated that women were more likely to have a lower mean MFR and an MFR value equal to or less than 2 compared with men; as well as that when adjusted for age, women with a score of equal to or less than 2 were more likely to experience adverse major cardiac events (MACE). A subsequent study that included patients without demonstrable coronary artery disease who underwent PET MPI confirmed this finding. These results showed that MFR was higher in younger males compared with younger females and decreased with age, while rest and stress MBF were higher in females compared with males across all ages (96). This can be partially explained by the fact that females have higher resting heart rate, blood pressure, and stress LVEF compared with males.

PET has been reported to have higher diagnostic accuracy with regard to wall motion imaging when compared to CMR and echocardiography, which have lower sensitivity (97). PET is often preferred over single-photon emission computed tomography myocardial perfusion imaging (SPECT MPI) as it is more likely to detect obstructive CAD findings (increased total perfusion deficit and impaired MFR) especially in patients with known cardiometabolic disease (98). To take it one step further, a study by Pelletier-Galarneau et al. found that patients with suspected CAD who underwent SPECT MPI were more likely to undergo coronary angiography, percutaneous coronary intervention (PCI) or coronary artery bypass grafting (CABG) than patients undergoing CCTA and PET MPI, while patients undergoing PET MPI as the modality of initial diagnosis for CAD exhibited a lower use of additional healthcare resources and had fewer hospital inpatient, outpatient and emergency department encounters compared with patients undergoing SPECT MPI, CCTA or stress echocardiography (99). This suggests that given its lower spatial and temporal resolution as well as sensitivity and diagnostic accuracy, SPECT MPI might lead to more unnecessary downstream invasive procedures and imaging that could compromise healthcare resource utilization and spending.

This contrasts with a prior study by Mullani et al. that compared patients who were randomly assigned to receive PET or SPECT (100). The study found no statistically significant difference in the number of positive scans by SPECT or PET, especially in patients with a documented history of CAD. Nonetheless, they did find that men compared with women, regardless of the presence of heart disease, were more likely to have a positive scan, consistent with the fact that men are more likely to present with obstructive lesions. Thus, the main argument circles back to the advantages of PET over SPECT, with the latter employing longer protocols with higher radiation dose, and with the former being able to identify non-obstructive phenotypes of CAD (i.e., diffuse atherosclerosis and microvascular dysfunction) and quantify MBF and MFR that can help determine which patients are at a higher risk of future cardiac events (101). However, PET MPI is more expensive and not widely available, while SPECT MPI also offers the advantages of easily combining with exercise stress if indicated.

## 8 Sex-specific differences identified by stress testing performance

Cardiac stress testing remains a cornerstone in guiding diagnosis for suspected CAD, risk stratification, and downstream management. Testing modalities include stress electrocardiogram, stress echocardiogram, PET or SPECT MPI, and stress cardiac MRI. However, a one-size-fits-all approach to cardiac stress testing evaluation tends to overlook important sex-specific differences that affect diagnostic accuracy and risk stratification. The WOMEN trial, a multicenter randomized clinical study compared exercise treadmill test (ETT) versus exercise SPECT MPI for suspected coronary artery disease in women, assessing whether the simpler lower cost EKG approach could safely replace nuclear imaging in women capable of exercising (102). The study found that women who underwent exercise EKG often achieved lower metabolic equivalents of task (METs), which in turn led to more test results being classified as abnormal or indeterminate (102). This study highlighted that compared with stress EKG, MPI was associated with greater initial costs, while the downstream procedural costs were only marginally lower despite no difference in MACE at the 2-year follow-up. The authors recommended that in low-risk symptomatic women with suspected CAD, ETT with selective follow-up testing is an appropriate diagnostic strategy.

Beyond their effects on lipid metabolism and vascular tone, hormones, such as estrogen, may also influence cardiac electrophysiology. Hormonal effects can be seen as measurable differences in ST-segment dynamics during stress testing. Estrogen has been reported to influence repolarization through alterations of ventricular repolarization leading to altered trafficking and function of potassium channels, contributing to higher baseline ST-T abnormalities in women and a greater rate of non-ischemic ST depressions (103). A study by Bokhari et al. evaluated 140 postmenopausal women (31 not taking any hormone replacement therapy [HRT], 75 taking estrogen alone [ERT], and 34 taking estrogen plus progesterone [EPRT]) who underwent exercise EKG followed by stress-gated MPI (104). Stress EKGs were considered positive if there was horizontal or down sloping ST-segment depression of at least 1 mm or an upsloping ST-segment depression of at least 1.5 mm at 80 ms after the J point compared with the baseline EKG. When comparing stress EKG results to gated MPI, no significant difference was noted in the sensitivity of detecting CAD in women among those on ERT (56%), EPRT (57%), or those not taking HRT (54%), the specificity of the women taking EPRT (80%) or those not taking HRT (78%) was significantly higher than those in women taking estrogen alone (46%). No significant differences were seen in peak heart rate and systolic blood pressure levels among the three groups. These observations highlight that estrogen therapy alone can increase the rates of false-positive stress EKG results. Similarly, another study by Henzlova et al. found that exercise-induced EKG positivity (defined as 1 mm or greater horizontal and/or down-sloping ST depression during and/or after exercise) in female patients who underwent gated SPECT was greater in postmenopausal women on HRT compared with premenopausal or postmenopausal females not on HRT (105).

To counter the false positive results obtained in females undergoing exercise EKG stress testing, stress echocardiography has emerged as a complementary imaging modality (106). Kim et al. evaluated 202 female patients who had a history of typical or atypical chest pain or ischemic equivalent symptoms and who underwent stress EKG or dobutamine stress echocardiography (DSE) one month prior to undergoing coronary angiography (107). The results showed that DSE was associated with greater sensitivity and specificity (70.4% and 94.6%, respectively) than treadmill exercise EKG testing (53.7% and 73.6%, respectively) for the detection of >50% coronary artery stenosis (CAS), along with greater accuracy in diagnosing coronary stenosis by either ≥50% and ≥75% CAS even in patients with intermediate pretest probability. These findings were corroborated in a meta-analysis which showed that DSE compared to stress nuclear scintigraphy was as sensitive and more specific to detect CAD, and that DSE in both men and women produced nearly identical sensitivity and specificity (108, 109). It is important to note however that false-positive test results have been reported in stress echocardiography as seen in the study by Keller et al. who evaluated male and female patients with suspected or known stable CAD who underwent bicycle-exercise stress echocardiography (110). Nonetheless, both exercise and dobutamine stress echocardiography methods have been found to be more specific than exercise EKG stress testing and equally sensitive and specific when compared with each other (111, 112).

Sex-specific differences have been reported in both stress CMR and stress SPECT modalities. Heydari et al. reported that among patients with suspicion of ischemic heart disease who underwent stress CMR, although women were more likely to have normal stress CMR findings compared with men, the primary (a composite of cardiovascular death and nonfatal myocardial infarction) and secondary (hospitalization for unstable angina or heart failure, and late unplanned coronary artery bypass grafting surgery) outcomes were largely non-significant between the two sexes (113). Nonetheless, women with abnormal CMR findings were more likely to experience higher rates of cardiac death or nonfatal myocardial infarctions compared with women with normal tests. In addition, Majstorov et al. found that among patients undergoing 99m-Tc sestamibi gated SPECT, women exhibited a significantly lower overall regional sensitivity (71.4% vs. 92.7%, *p* = 0.039) compared with men, however no significant differences in sensitivity were detected among the three vascular territories (left anterior descending, left circumflex and right coronary arteries) (114). The authors did not find any significant difference in overall regional specificity, with the only exception being a significantly lower specificity (79.1% vs. 100%) in the right coronary artery territory for men compared with women. While the above sex-specific differences in findings of PET and SPECT MPI have been documented, somewhat discrepant findings have been reported in studies comparing stress CMR with SPECT MPI. Reports have suggested that stress CMR is associated with greater specificity and accuracy compared to SPECT MPI albeit with no difference in sensitivity (115). Others studies have found that CMR adenosine stress/rest perfusion, cine imaging, late gadolinium enhancement, and magnetic resonance coronary angiography) compared to gated adenosine stress/rest SPECT is associated with greater sensitivity and similar specificities in both sexes (116). A summary of the above modalities are provided in Table 1.

**Table 1:** Sex differences compared among stress testing modalities (62, 102, 117–123).

Sex differences in stress testing modalities				
Attribute	Stress EKG	Stress echocardiography	Myocardial perfusion imaging (SPECT/PET)	Stress cardiac magnetic resonance (CMR)
Diagnostic Accuracy	Lower in women due to lower peak workloads and more false positives	Comparable in women and men; higher sensitivity/specificity than exercise EKG in women	Comparable in women and men; PET has higher accuracy compared to SPECT	Equal performance in women and men; high accuracy observed in women at intermediate-to-high risk for CAD
Sex-specific considerations	- Hormonal fluctuations may affect EKG interpretation - Lower peak workloads may reduce sensitivity	- No radiation - Useful for valvular disease and pulmonary hypertension - Operator-dependent	- Radiation exposure higher in women - Breast attenuation artifacts - Smaller heart size affects image quality	- No radiation - High spatial resolution - Assesses myocardial fibrosis and infiltration - Limited availability
Clinical outcomes	Similar prognostic values in women and men when adjusted for pretest probability	Normal results indicate low risk; similar prognostic values in women and men	Similar prognostic values in women and men; severe defects carry higher mortality risk	Similar prognostic values and rates of MACE in women and men diagnosed with ischemia

CAD, cardiovascular atherosclerotic disease; MACE, major adverse cardiovascular events.

## 9 Conclusions and why understanding these sex-specific differences matters

In summary, understanding that the female heart isn't just a smaller version of the male one is crucial. The differences that have been identified in cardiac imaging studies can allow one to better understand how cardiovascular disease manifests and progresses in females. The presence of LV stiffness in females which increases with aging, in conjunction with LV concentric remodeling and arterial stiffness, predisposes women to the formation of diastolic dysfunction and HFpEF (124). Indeed, coronary microvascular dysfunction (CMD) is present in most patients with HFpEF, with myocardial blood flow (MBF) and coronary flow reserve (CFR) being key elements of CMD (125). PET MPI has demonstrated that MBF values at rest are higher and CFR values are lower in women than in men. While a possible explanation for the higher MBF values in women could be the increase in rest cardiac metabolic demand, studies have shown that higher rest MBF values are associated with worse diastolic function (higher E/e′) (126). Lower CFR in the setting of a higher rest MBF can further predispose patients to remodeling of the microvasculature, altered endothelial function, and smooth muscle dysfunction, making it an integrated marker of coronary vasomotor dysfunction (119).

Similarly, echocardiography and CMR imaging have improved our understanding of valve lesions in women, especially AS. Indeed, women are more predisposed to the development of low-flow low-gradient AS compared with men (88). Patients with low-flow low-gradient AS are at greater risk for worse clinical outcomes, including cardiac events, all-cause mortality, and cardiovascular mortality (127). This is largely due to increased filling pressures in the setting of severe LV diastolic dysfunction despite the possible presence of a preserved ejection fraction, as well as due to the possible underestimation of AS severity given a very narrow valve area (128).

With regard to CAD, the use of CCTA has allowed us to see that men have higher average calcium scores in coronary arteries than females, along with a greater tendency to have multiple vessel involvement (129). This can partially be attributed to the fact that women have smaller epicardial arteries, higher coronary blood flow, and lower resulted endothelial shear stress, which can further explain the lower CAD rates in females (130). While ischemic heart disease (IHD) remains the leading cause of death in women, the presence of ischemia with no obstructive coronary arteries (INOCA) in women is also associated with significant cardiac mortality rates (131). This is not surprising, as two of the primary mechanisms of INOCA include CMD and coronary vasospasm. Moreover, the high rates of false positive tests in women undergoing exercise EKG testing further highlights interpretation challenges, especially in women on HRT who may present with more ST-segment depression during exercise testing, a finding that can mimic INOCA (104).

It is not only the presence or underdiagnosis of certain conditions, such as CMD, INOCA or low-flow low-gradient AS that predispose women to worse clinical outcomes. For example, women are also less likely than men to receive guideline-recommended medications, such as statins, beta blockers or antiplatelet therapies (130). Moreover, in addition to being offered fewer diagnostic tests, women are also less likely to receive cardiac interventions and be referred for cardiac rehabilitation (132, 133). Therefore, in an era where patient-centered care is emphasized, it will be important to modify guidelines as needed to ensure that women, like men, also receive all indicated and appropriate treatments and interventions for their cardiac conditions and prevention. By doing so, gender disparities in cardiovascular care can be eliminated or minimized, especially in view of the increased burden that women may already face secondary to socioeconomic, racial, and cultural factors (132).

Lastly, understanding sex-specific differences in electrophysiology is of equal importance, with women tending to have longer corrected QT intervals that predispose them to a higher incidence of arrhythmic events, particularly torsade de pointes (32). Therefore, by combining all these findings and by appreciating sex-specific differences in cardiac anatomy and function, we can not only help reduce systematic biases in healthcare and avoid under-treating cardiovascular pathologies in women, but can further create guidelines to recommend the use of modalities that can better detect CMD (for example with CMR). In addition, by recognizing certain observed sex-specific differences in cardiac imaging we can better discern the prevalence of certain conditions such as HFpEF and diffuse atherosclerosis and CMD in women. It is only a matter of time before everyone can truly say that the female heart has the same shape but a different story than the male one.
